# Concomitant Single-Stage Unifocalization and Cavopulmonary Anastomosis (Glenn Shunt) in an Adolescent Patient With Univentricular Physiology and Major Aortopulmonary Collateral Arteries: A Technically Challenging Case

**DOI:** 10.7759/cureus.20260

**Published:** 2021-12-08

**Authors:** Vishal V Bhende, Hardil P Majmudar, Tanishq S Sharma, Varin Rangwala, Viral B Patel, Amit Kumar, Gurpreet Panesar, Sohilkhan R Pathan, Saptak P Mankad

**Affiliations:** 1 Pediatric Cardiac Surgery, Bhanubhai and Madhuben Patel Cardiac Centre, Shree Krishna Hospital, Anand, IND; 2 Cardiac Surgery, Bhanubhai and Madhuben Patel Cardiac Centre, Shree Krishna Hospital, Anand, IND; 3 Internal Medicine, Shree Krishna Hospital, Anand, IND; 4 Radiodiagnosis, Pramukhswami Medical College and Shree Krishna Hospital, Bhaikaka University, Karamsad, Anand, IND; 5 Pediatric Cardiology, Bhanubhai and Madhuben Patel Cardiac Centre, Shree Krishna Hospital, Anand, IND; 6 Cardiac Anaesthesia, Bhanubhai and Madhuben Patel Cardiac Centre, Shree Krishna Hospital, Anand, IND; 7 Clinical Research, Bhanubhai and Madhuben Patel Cardiac Centre, Shree Krishna Hospital, Anand, IND

**Keywords:** pediatric cardio thoracic surgery, congenital heart surgery, ventricular septal defect (vsd), double inlet ventricle or single ventricle or univentricular heart, bidirectional glenn procedure, unifocalization

## Abstract

Long-segment pulmonary atresia (PA), non-confluent branch pulmonary arteries, ventricular septal defect, tricuspid valve atresia (type 1A), and single ventricle physiology is a relatively rare and extremely heterogeneous form of congenital heart disease. This subset of patients having pulmonary atresia, ventricular septal defect, and major aortopulmonary collateral arteries (MAPCAs) have to undergo multiple unifocalization staging operations before a complete repair is attempted. Most of the patients were deemed inoperable. We report a rare case of a concomitant single-stage unifocalization and cavopulmonary anastomosis (bi-directional Glenn procedure) in an adolescent cyanotic girl with tricuspid valve atresia (type 1 A), long-segment pulmonary atresia, non-confluent branch pulmonary arteries, bilateral patent ductus arteriosus, MAPCAs, and single-ventricle physiology. Reconstruction of the absent central pulmonary artery and non-confluent branch pulmonary arteries was achieved by dividing the bilateral patent ductus arteriosus feeding the bilateral pulmonary arteries.

## Introduction

Functional single ventricle and major aortopulmonary collateral arteries (MAPCAs) still remain a surgical challenge in low-resource settings [1]. While still there is no harmony in the perfect treatment for these anomalies. We report a successful concomitant single-stage unifocalization and bidirectional Glenn procedure in a 10-year-old child in a rural tertiary care setting.

## Case presentation

We report a case of a 10-year-old girl weighing 20.7 kg at the time of surgery who presented with complex cyanotic congenital heart diseases (late presentation) - tricuspid valve atresia (type 1-A), restricted atrial septal defect (ASD), large ventricular septal defect, long segment pulmonary atresia (PA), non-confluent branch pulmonary arteries, hypoplastic right ventricle, dominant left ventricle, MAPCAs, and single-ventricle physiology - was referred to our cardiac center. She complained of breathlessness and fatiguability gradually increased over time and which were aggravated by physical activity and relieved by rest. On examination, she had poor weight gain since birth and dyspnea on exertion (NYHA Grade 3) for five years. The patient had central as well as peripheral cyanosis and clubbing.

**Figure 1:**
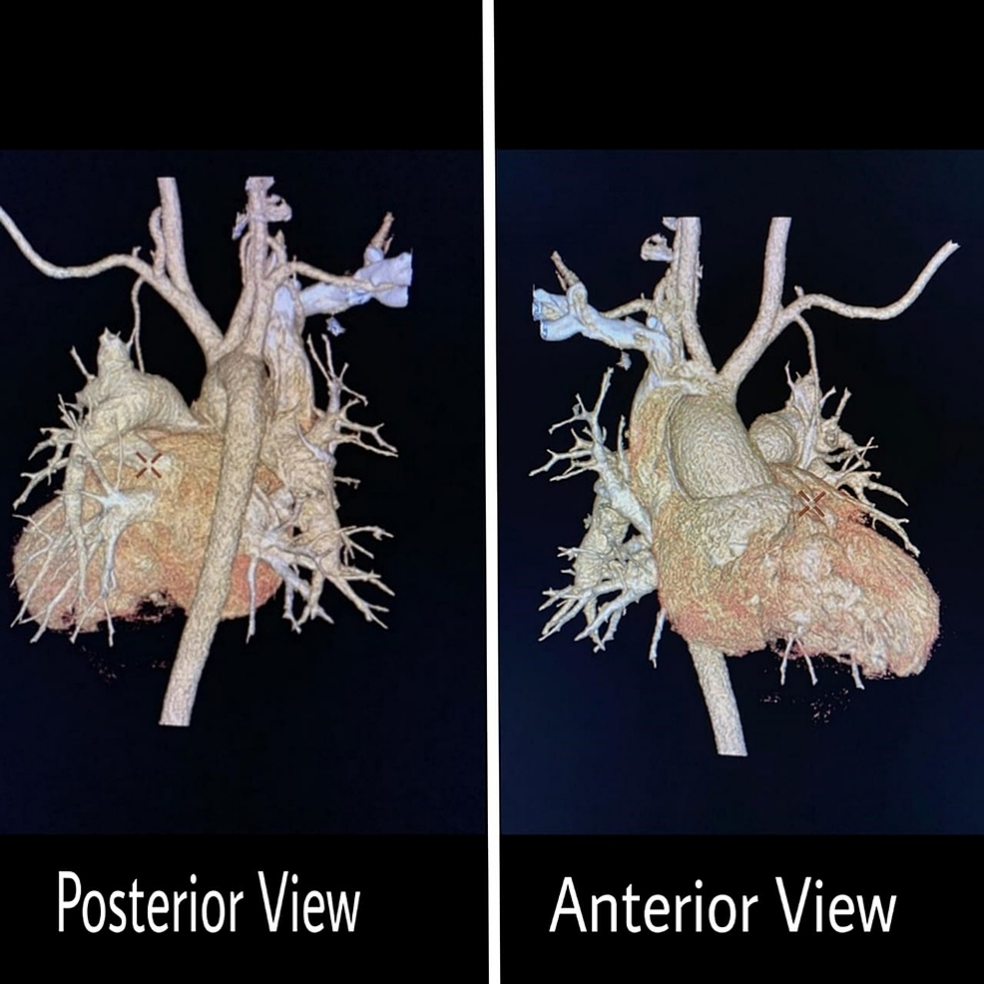
Volume rendering CT cardiac dynamic study showing a posterior and anterior view of the heart. Image credits: Dr. Viral B. Patel.

**Figure 2:**
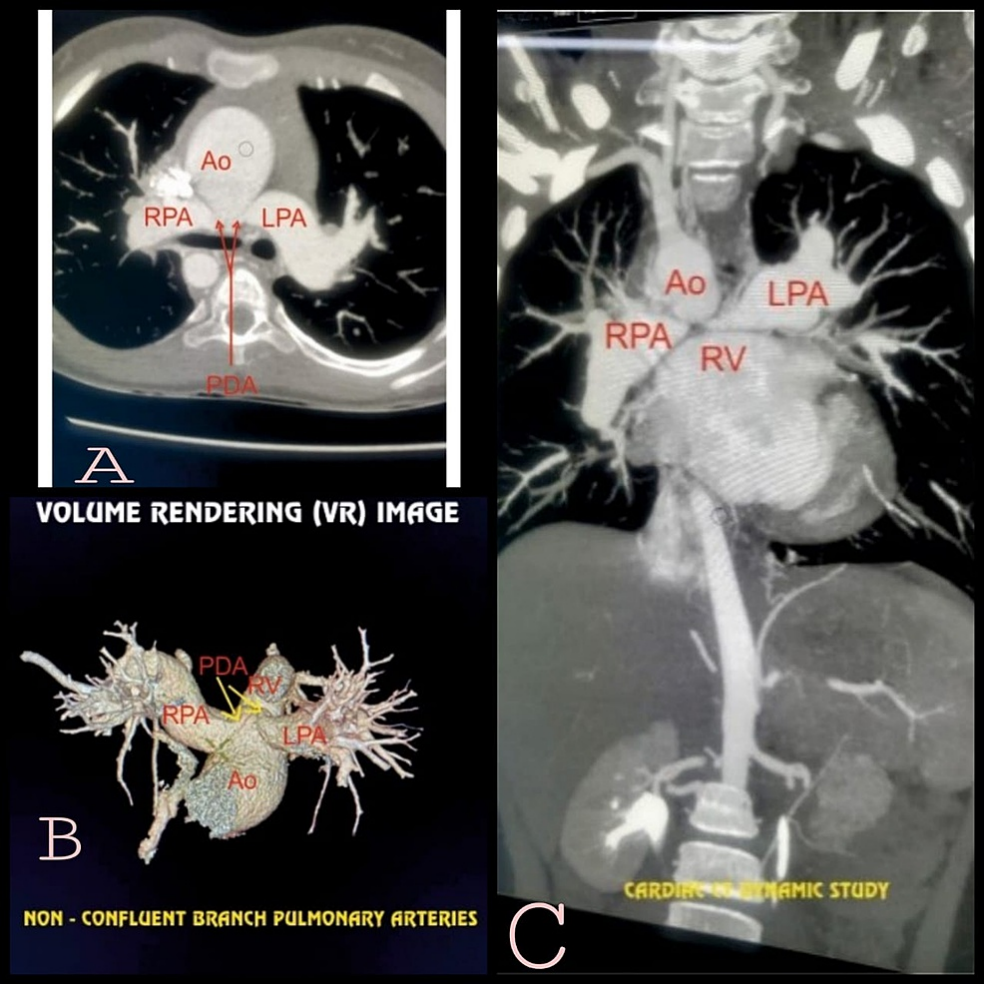
Volume rendering CT cardiac dynamic study showing non-confluent branch pulmonary arteries. Image credits: Dr. Viral B. Patel.

She underwent detailed cardiological evaluation with 2D echocardiography, cardiac computed tomography dynamic study. Cardiac CT dynamic study revealed that the branch pulmonary arteries were non-confluent and had feeders from the aortic arch through short bilateral patent ductus arteriosus (Figures 1-2). 

**Figure 3:**
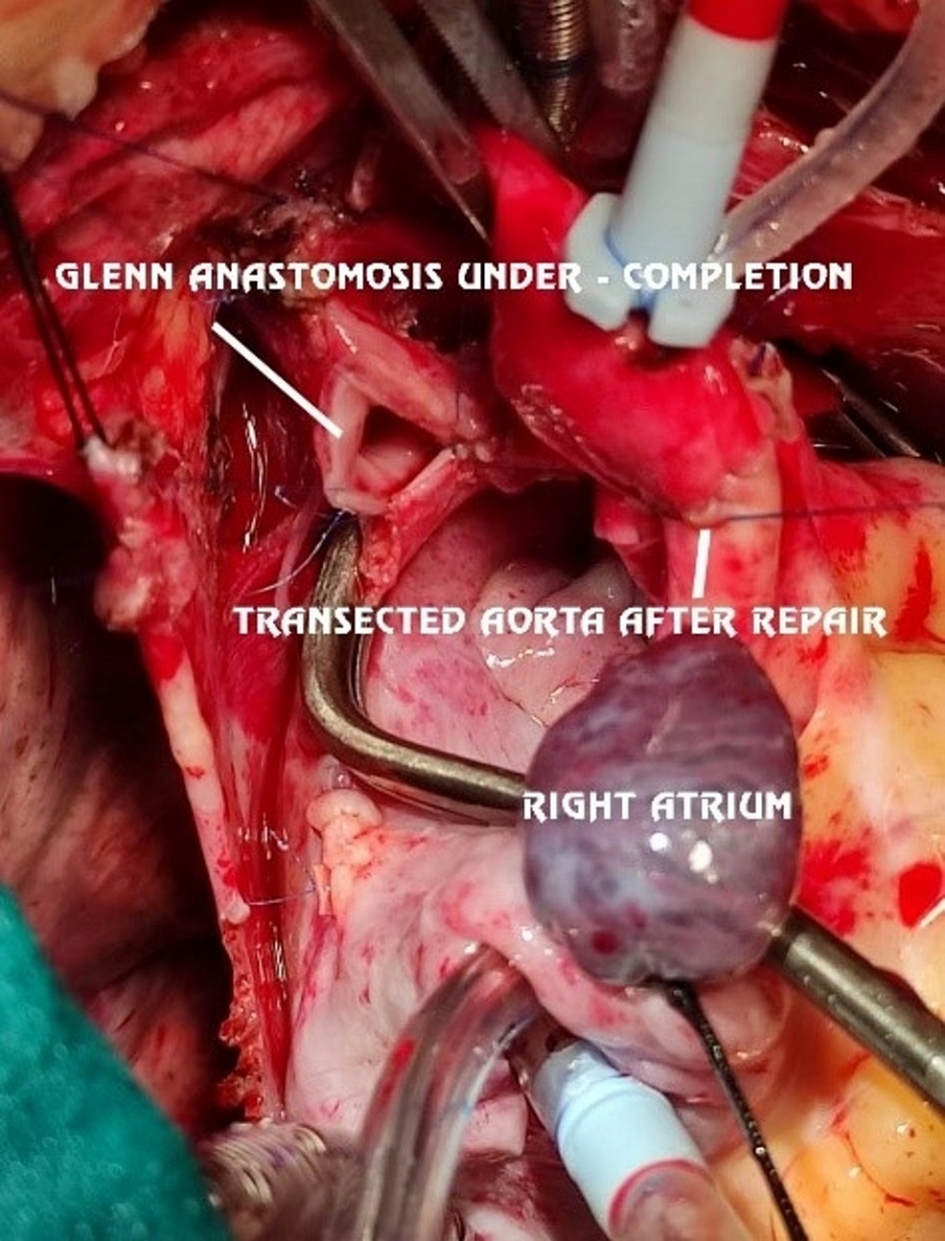
Glenn anastomosis commencement after unifocalization completion. Image credits: Dr. Vishal Bhende.

**Figure 4:**
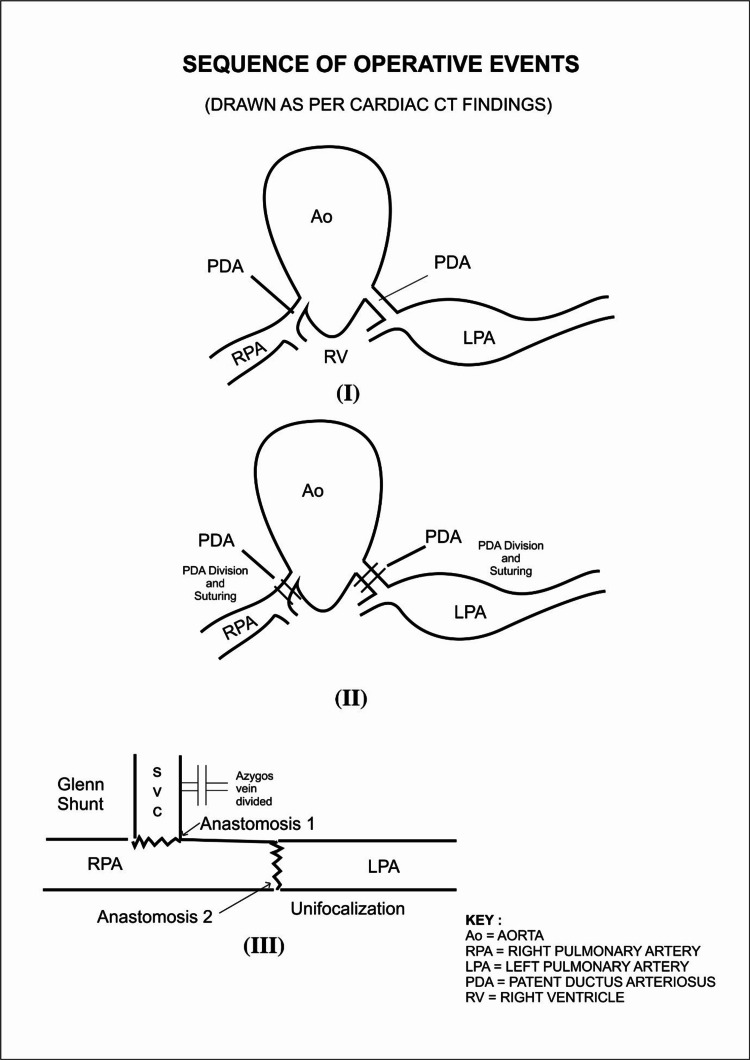
Diagrammatic representation of a sequence of operative events. Diagram credits: Dr. Vishal Bhende.

To plan further palliation, it was necessary to record pressure in the individual pulmonary arteries and it was attempted by pre-operative angiography/catheterization studies. Selective cannulation of collateral arteries/bilateral ductus was tried multiple times to enter pulmonary arteries but was not successful, and hence, the procedure was abandoned. With written informed high-risk consent and Covid-19 (RT-PCR) reports negative, the patient underwent complete single-stage unifocalization and concomitant bi-directional Glenn procedure with division and suturing of bilateral patent ductus arteriosus and atrial septectomy (Figures 3-4). The entire surgical procedure was done on the cardio-pulmonary bypass (CPB) and after ascertaining invasive pressures in RPA:23/17(20) mmHg; LPA:15/10(11) mmHg to be suitable for palliation. The surgical summary of steps is given in Table 1.

**Table 1:** The surgical procedure and summary of steps. RPA: right pulmonary artery, LPA: left pulmonary artery.

Perfusion details	
	Myocardial protection – hypothermia 24 °C
	Total circulatory arrest time: 44 minutes
	Cardiopulmonary bypass time: 284 minutes
	Aortic cross-clamp time: 218 minutes
Surgical details	
	Aortic transection above the level of coronary Ostia to facilitate mobilization of non-confluent branch pulmonary arteries.
	Mobilization of the right pulmonary artery and left pulmonary artery up to the respective hila.
	Unifocalization done between the RPA and LPA after dividing the bilateral ductus arteriosus. This reconstruction of the central portion of branch pulmonary arteries was done using 6-0 polypropylene continuous sutures.
	The restricted atrial septal defect was enlarged. Atrial septectomy after opening the right atrium.
	Bi-directional Glenn shunt was performed by using the superior vena cava and the azygos vein was divided.

**Figure 5:**
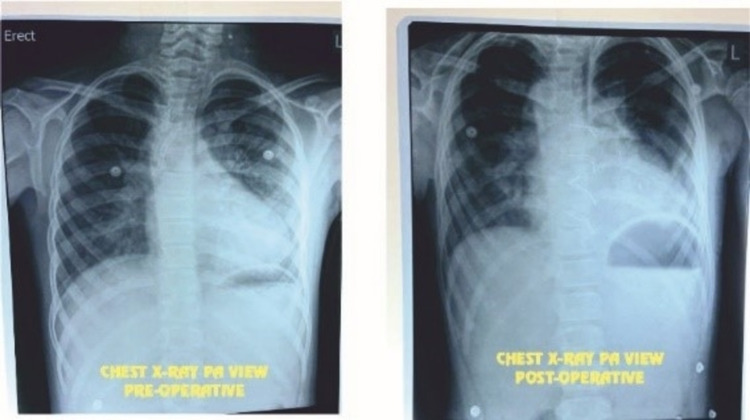
Preoperative and postoperative chest X-rays (PA view).

The patient was weaned off from the CPB and delayed chest closure was undertaken on postoperative day (POD) 2 for better surgical results and to overcome myocardial edema. She had a stormy postoperative period with hemodynamic instability needing cardiopulmonary resuscitative (CPR) measures twice and hiking up of inotropic supports. She was extubated on a POD 6 after 168 hours. Oral feeds were started gradually and she was discharged in a stable condition and healthy wound. Her hemoglobin is 11.6 g/dl, total leucocyte count is 16.6 × 1000/µl, and SPO_2_ is 72-79% on discharge. Regular follow-up 2D echocardiography revealed well flowing right bi-directional Glenn shunt without any turbulence; good flow in the RPA and LPA: RPA 12 mm and LPA 9.4 mm with saturations ranging between 72% and 79%. Post-operative X-ray revealed no abnormalities (Figure 5). 

## Discussion

Functionally single-ventricle physiology and MAPCAs highlight a significantly heterogeneous subset of patients. Though the mortality and morbidity remain high most of them can benefit from elective palliative surgery by eventually completing the Fontan circulation [1,2]. Complete unifocalization with good peripheral pulmonary arteries are the determining factors for successful palliation in this subset of patients [1].

Pulmonary atresia with MAPCAs and single-ventricle physiology still remains a rare subset. MAPCAs, as an abnormality, are rather infrequent in practice. Rarely, case studies and series of patients with single ventricle and MAPCAs have been reported in the past. Three percent of all patients with right isomerism present with MAPCAs [3]. However, in the literature, only a single case report of a patient with MAPCAs, good-sized central pulmonary arteries, and heterotaxy/asplenia/unbalanced atrioventricular canal, who was unifocalized and a complete Fontan circulation was achieved, is reported [4]. A patient with heterotaxy and unbalanced atrioventricular canal with a single MAPCA, who was unifocalized and a cavopulmonary shunt was performed eventually, was described by Ko et al. in their report from Japan [5]. Single-ventricle patients with MAPCAs are a non-ubiquitous group. There are seldom a few reports of patients achieving complete cavopulmonary anastomosis. Compulsory pulmonary blood flow, lower risk of thrombosis compared to aortopulmonary shunts, and reduced volume load and incidence of heart failure can be facilitated by a cavopulmonary anastomosis and it has proven to be cardio-protective in this subset of patients [6,7].

Concomitant cavopulmonary anastomosis was performed owing to the fact that complete unifocalization was achieved in a very young patient with good perfusion in peripheral pulmonary arteries. Superior vena cava pressure was 15 mmHg after surgery and remained constant on follow-up. Stent implantation in highly stenotic MAPCAs to ensure adequate perfusion to the lung is an alternative in dealing with highly stenotic MAPCAs; in younger patients can be managed by stent implantation as an alternative where the pulmonary artery development cannot be bargained. What makes it technically challenging is the rare absence of central pulmonary arteries and it is very essential to provide adequate growth potential to the newly reconstructed pulmonary artery [8]. Our patient responded well and we attribute it to the significant avoidance of any foreign materials in reconstruction. Good exposure for reconstructing the central pulmonary artery is possible via mobilization of the ascending aorta and aortic arch. For mobilization and adequate exposure of the pulmonary arteries, division of the ascending aorta is crucial [9]. Abnormalities in the coronary arteries attribute an additional risk factor in patients with pulmonary atresia with the intact ventricular septum (PAIVS) in addition to several other factors (Table 2). This risk is consistently higher than that seen in single ventricle physiology. Therefore, it is essential to seek abnormal coronaries in all patients of PAIVS and to exclude the additional risk for performing unifocalization in all infants [10].

**Table 2:** Risk factors in consideration for unifocalization. PAIVS: Pulmonary atresia with the intact ventricular septum, TAPVR: total anomalous pulmonary venous return, MAPCAs: major aortopulmonary collateral arteries.

Risk factors in consideration for unifocalization
PAIVS
TAPVR + MAPCAs
Cor-triatriatum + MAPCAs
Tracheobronchomalacia
Heterotaxy+Asplenia is a partial risk factor

## Conclusions

The combination of pulmonary atresia, tricuspid atresia, MAPCAs with single-ventricle physiology is found to be an extremely rare presentation accounting for only a small percentage of all patients with MAPCAs. Most of these patients have been considered inoperable and the mortality is significant, which confirms that this group is a very high-risk group. On the other hand, we achieved a good pulmonary vascular bed by recruiting branch pulmonary arteries and achieved a low pulmonary vascular resistance in our patient to perform bidirectional Glenn shunt at this stage. Adequate counseling for the parents needs to be sought at every step in the process and the significant risk of death owing to coronary problems needs to be explained clearly. As per the literature, concomitant single-stage unifocalization and cavopulmonary anastomosis (Glenn shunt) should be contemplated in patients with univentricular physiology and tricuspid atresia and the pulmonary vascular bed can be achieved by unifocalization.
